# Changes in Epicardial Adipose Tissue Assessed by Chest CT in Breast Cancer Patients Receiving Adjuvant Chemotherapy with Anthracyclines and Trastuzumab

**DOI:** 10.31083/j.rcm2507254

**Published:** 2024-07-09

**Authors:** Yuyao Liu, Tingjian Zhang, Xiao Huang, Li Shen, Quan Yang

**Affiliations:** ^1^Department of Radiology, Yongchuan Hospital of Chongqing Medical University, 402160 Chongqing, China; ^2^Department of General Surgery, Yongchuan Hospital of Chongqing Medical University, 402160 Chongqing, China

**Keywords:** cardiotoxicity, epicardial adipose tissue, breast cancer, anthracyclines, trastuzumab

## Abstract

**Background:**

Cardiotoxicity (CTX) induced by adjuvant chemotherapy is a significant factor that impacts the prognosis and quality of life in breast cancer (BC) patients. In this study, we aimed to investigate the changes in epicardial adipose tissue (EAT) before and after treatment in BC patients who received anthracyclines adjuvant chemotherapy protocol (AC-T) and anthracyclines combined with trastuzumabadjuvant chemotherapy protocol (AC-TH). Additionally, we assessed whether there were any differences in the changes in EAT between the two groups of patients. Our objective was to examine the effects of anthracyclines and trastuzumab on EAT and determine the potential role of EAT changes on CTX.

**Methods:**

We reviewed female BC patients who were treated with adjuvant chemotherapy protocols of AC-T and AC-TH, all of whom underwent baseline (T0) and follow-up (T1) chest computed tomography (CT) and echocardiography. A cohort of healthy women, matched in age, underwent two chest CTs. EAT was quantified on chest CT using semi-automated software. CTX was defined as a >10% reduction in left ventricular ejection fraction (LVEF) from baseline, with an absolute value of <53%.

**Results:**

A total of 41 BC patients were included in the study, with 23 patients in the AC-T group and 18 patients in the AC-TH group. Additionally, 22 healthy females were included as the normal group. None of the BC patients developed CTX after chemotherapy. The age did not differ significantly between the normal group and the AC-T group (*p* = 0.341) or the AC-TH group (*p* = 0.853). Similarly, the body mass index (BMI) of the normal group was comparable to that of the AC-T group (*p* = 0.377, 0.346) and the AC-TH group (*p = *0.148, 0.119) before and after chemotherapy. The EAT volume index (mL/kg/$m^{2}$) was significantly higher in both the AC-T group (5.11 ± 1.85 vs. 4.34 ± 1.55, *p* < 0.001) and the AC-TH group (4.53 ± 1.61 vs. 3.48 ± 1.62, *p* < 0.001) at T1 compared with T0. In addition, both the AC-T group (–72.95 ± 5.01 vs. –71.22 ± 3.91, *p* = 0.005) and the AC-TH group (–72.55 ± 5.27 vs. –68.20 ± 5.98, *p* < 0.001) exhibited a significant decrease in EAT radiodensity (HU) at T1 compared to T0. However, there was no significant difference observed in the normal group. At T0, no difference was seen in EAT volume index (4.34 ± 1.55 vs. 3.48 ± 1.62, *p* = 0.090) and radiodensity (–71.22 ± 3.91 vs. –68.20 ± 5.98, *p* = 0.059) between the AC-T and AC-TH groups. Similarly, at T1, there was still no significant difference observed in the EAT volume index (–5.11 ± 1.85 vs. 4.53 ± 1.61, *p* = 0.308) and radiodensity (–72.95 ± 5.00 vs. –72.54 ± 5.27, *p* = 0.802) between the two groups.

**Conclusions:**

BC patients who underwent AC-T and AC-TH adjuvant chemotherapy protocols demonstrated a significant rise in the volume index of EAT, along with a substantial reduction in its radiodensity post-chemotherapy. These findings indicate that alterations in EAT could potentially aid in identifying cardiac complications caused by chemotherapeutic agents and remind clinicians to focus on changes in EAT after adjuvant chemotherapy in BC patients to prevent the practical occurrence of CTX.

## 1. Introduction

Breast cancer (BC) has become the most commonly diagnosed cancer and the leading cause of cancer death in women surpassing lung cancer [1]. Anthracyclines are a mainstay adjuvant treatment for patients with advanced disease or triple-negative neoplasms, and they significantly reduce the 10-year risk of breast cancer recurrence and mortality [2]. In addition, the rate of human epidermal growth factor receptor 2 (HER2) positivity is 15% to 30% and is generally associated with a poor prognosis [3]. Trastuzumab, the first humanized monoclonal antibody targeting HER2, significantly improved clinical outcomes in HER2-positive patients [4]. However, Cardiotoxicity (CTX) is a well-known complication associated with both anthracyclines and trastuzumab, and can determine the prognosis in cancer survivors [5]. Anthracyclines exert their effects through mechanisms such as topoisomerase inhibition and oxidative stress, which contribute to progressive myocardial fibrosis and are correlated with the cumulative dose of anthracycline [6]. In contrast, trastuzumab-induced cardiotoxicity is not dose-dependent and is reversible, and it is thought to occur through inhibition of the Neuregulin-1 (NRG-1)/HER and downstream signaling pathway [7].

Epicardial adipose tissue (EAT) is a type of visceral adipose tissue that is found between the myocardium and the visceral layer of the epicardium. It plays a crucial role in regulating metabolic activity in the surrounding area through paracrine signaling. Consequently, alterations in EAT are believed to be linked to a higher risk of adverse cardiovascular events [8, 9]. Measurement of EAT on the free wall of the right ventricular using echocardiography was the first technique used for quantification of EAT thickness. However, this method has limitations as it cannot accurately measure the entire volume of EAT and relies on the experience and technique of the operator. Cardiac magnetic resonance imaging (MRI) can accurately measure the volume of EAT without exposing patients to radiation, but is associated with high cost, long examination time, and can trigger claustrophobic events in patients, all of which limits its application in clinical practice [8, 10]. Chest computed tomography (CT) can image the entire heart to provide accurate EAT volumes and densities. Patients receiving BC adjuvant chemotherapy require regular follow-up with chest CT, so an additional risk assessment can be performed using EAT without additional radiation exposure or cost [11, 12]. A recent study has reported an increase in EAT radiodensity and volume in BC patients who received adjuvant chemotherapy with anthracyclines and trastuzumab [3, 12]. However, another study discovered a decrease in EAT radiodensity following adjuvant chemotherapy with anthracyclines [13].

Given the different mechanisms leading to CTX between anthracyclines and trastuzumab and the key role of EAT in adverse cardiovascular events, the objective of this study was to evaluate the alterations in EAT radiodensity and volume in BC patients treated with anthracyclines (AC-T) and anthracyclines combined with trastuzumab (AC-TH) adjuvant chemotherapy and to assess whether there was a difference in the changes in EAT radiodensity and volume between the two groups in order to understand the potential role of EAT changes in CTX.

## 2. Materials and Methods

### 2.1 Study Population

**Fig. 1.**
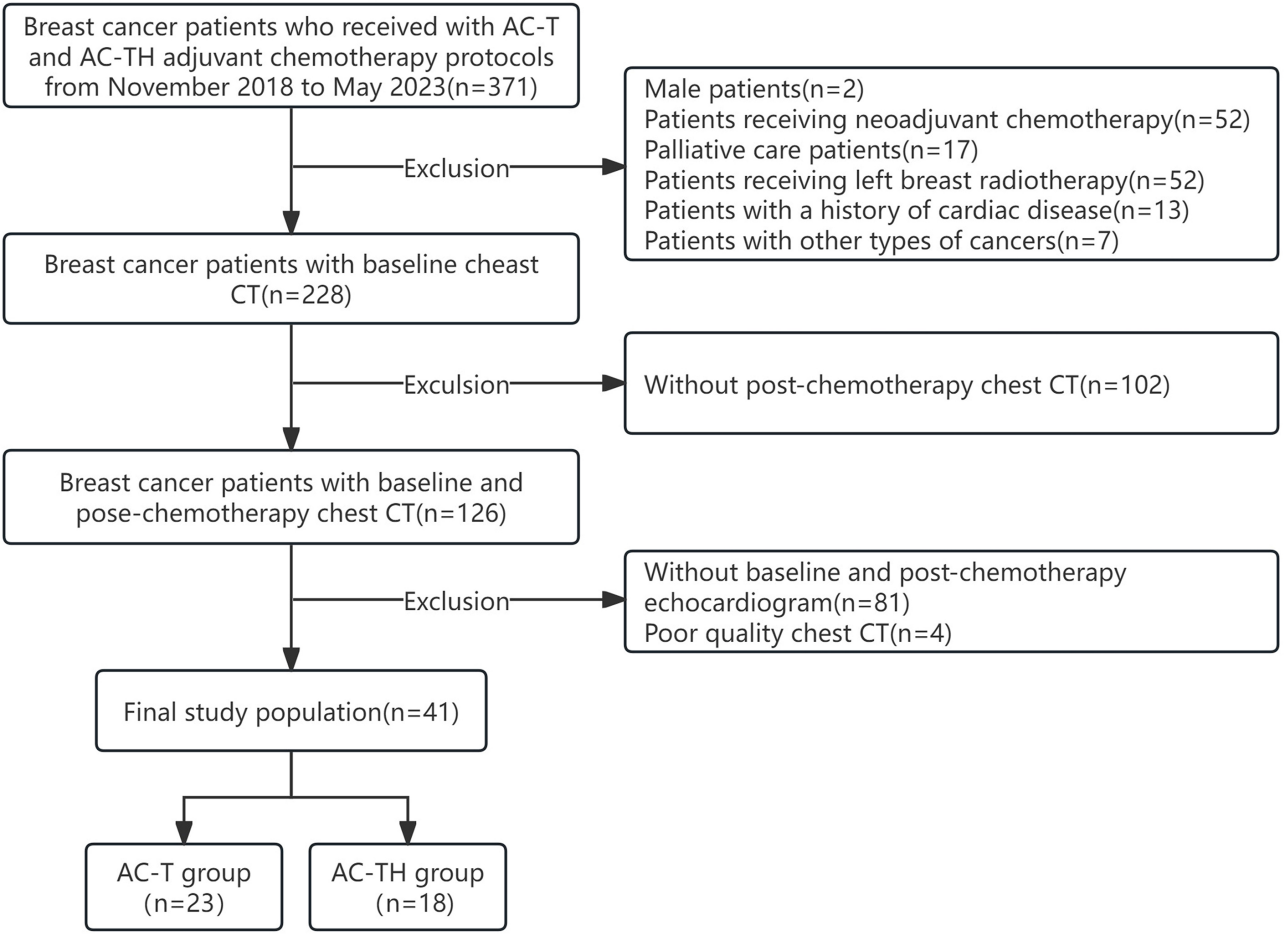
**Selection of the study population.** AC-T, anthracyclines adjuvant chemotherapy protocol; AC-TH, anthracyclines combined with trastuzumab adjuvant chemotherapy protocol; CT, computed tomography.

We recruited breast cancer patients who received adjuvant chemotherapy with anthracyclines and anthracyclines combined with trastuzumab between November 2018 and May 2023. During the treatment period, these patients underwent at least two chest non-enhanced CT scans to detect lung metastases and determine the stage of the disease. One of the scans was performed at the baseline before the start of chemotherapy (T0), and the other was a follow-up scan after the completion of chemotherapy (T1). The inclusion criteria for this study were as follows: (1) patients with pathologically confirmed, completely excised invasive BC; (2) patients undergoing adjuvant chemotherapy with anthracyclines and cyclophosphamide followed by docetaxel every 3 weeks (AC-T) or the same protocol plus 52 weeks of trastuzumab (AC-TH), in accordance with guidelines [11]; (3) patients who had at least two non-enhanced chest CT scans and echocardiograms, both before the start of chemotherapy and at the end of chemotherapy, and had a normal left ventricular ejection fraction (LVEF) at baseline. Exclusion criteria were as follows: (1) patients with a prior history of cardiac disease (e.g., myocardial infarction, heart failure, heart valve disease, cardiomyopathy, severe arrhythmia); (2) patients who had received radiotherapy for left BC or prior chemotherapy; (3) male patients or patients with cancer types other than BC; (4) patients with unclear imaging data or incomplete clinical data. Finally, 41 patients with BC treated with adjuvant chemotherapy were included. The flow diagram of participants is shown in Fig. 1.

To establish a control population, we enrolled age-matched women from the physical examination center during the same period. These women underwent two chest non-enhanced CTs, with an interval of approximately 18 months (similar to the interval observed in the group of patients receiving the AC-TH adjuvant chemotherapy protocol, which had a longer duration of chemotherapy). Participants with a history of cardiac disease, previous chemotherapy, radiotherapy, and any other cardiotoxic treatment were excluded. The study protocol adhered to the Declaration of Helsinki and was approved by the Ethics Committee of Yongchuan Hospital of Chongqing Medical University (No. 2023-025). Given the retrospective nature of this study, the requirement for informed consent was waived.

### 2.2 Chemotherapeutic Regimens

Patients in both groups received polychemotherapeutic protocols after BC surgery. For HER-2 positive BC patients, a standardized AC-TH protocol was used, consisting of anthracyclines (epirubicin: 90 ${\mathrm{mg/m}}^{2}$ or doxorubicin: 60 ${\mathrm{mg/m}}^{2}$ or doxorubicin liposomes: 20 ${\mathrm{mg/m}}^{2}$), cyclophosphamide (600 ${\mathrm{mg/m}}^{2}$ every 3 weeks for a total of four cycles, followed by docetaxel 100 ${\mathrm{mg/m}}^{2}$ every 3 weeks for four doses), along with trastuzumab (8 mg/kg as an initial loading dose, followed by 16 cycles of maintenance at 6 mg/kg, every 3 weeks). To ensure consistent usage of anthracyclines in both groups, the AC-T protocol was chosen for the polychemotherapeutic protocols in the other group of patients (same as the AC-TH protocol but without trastuzumab treatment). In order to minimize the risk of nausea and vomiting caused by anthracycline and cyclophosphamide combination chemotherapy regimens, each patient was given a 5-hydroxytryptamine-3 receptor antagonist (5-HT3RA) and a single dose of dexamethasone before receiving these two drugs to prevent vomiting.

### 2.3 CT Protocol

Chest CT examinations were performed without contrast in a scanner (Brilliance iCT, Philips Medical Systems, Cleveland, OH, USA). All patients were scanned from the apices to the base of the lungs using a peak voltage of 120 kVp and an automatic tube current (50–300 mAs) while holding their breath in the supine position. All scans were reconstructed using high spatial frequency, iterative reconstruction (iDose 4, Philips Medical Systems, Cleveland, OH, USA) with a slice thickness of 1 mm as axial images. All patients were examined using echocardiography (GE Voluson E6, GE Healthcare Austria GmbH & Co OG, Tiefenbach, Zipf, Austria or Canon APLIO 500 TUS-A500, Canon Medical Systems Inc., Shimoishigami, Otawara-shi, Tochigi, Japan) before and at the end of chemotherapy, LVEF was measured using the biplane Simpson method according to the guidelines of the American Society of Echocardiography [14].

### 2.4 Quantification of EAT

**Fig. 2.**
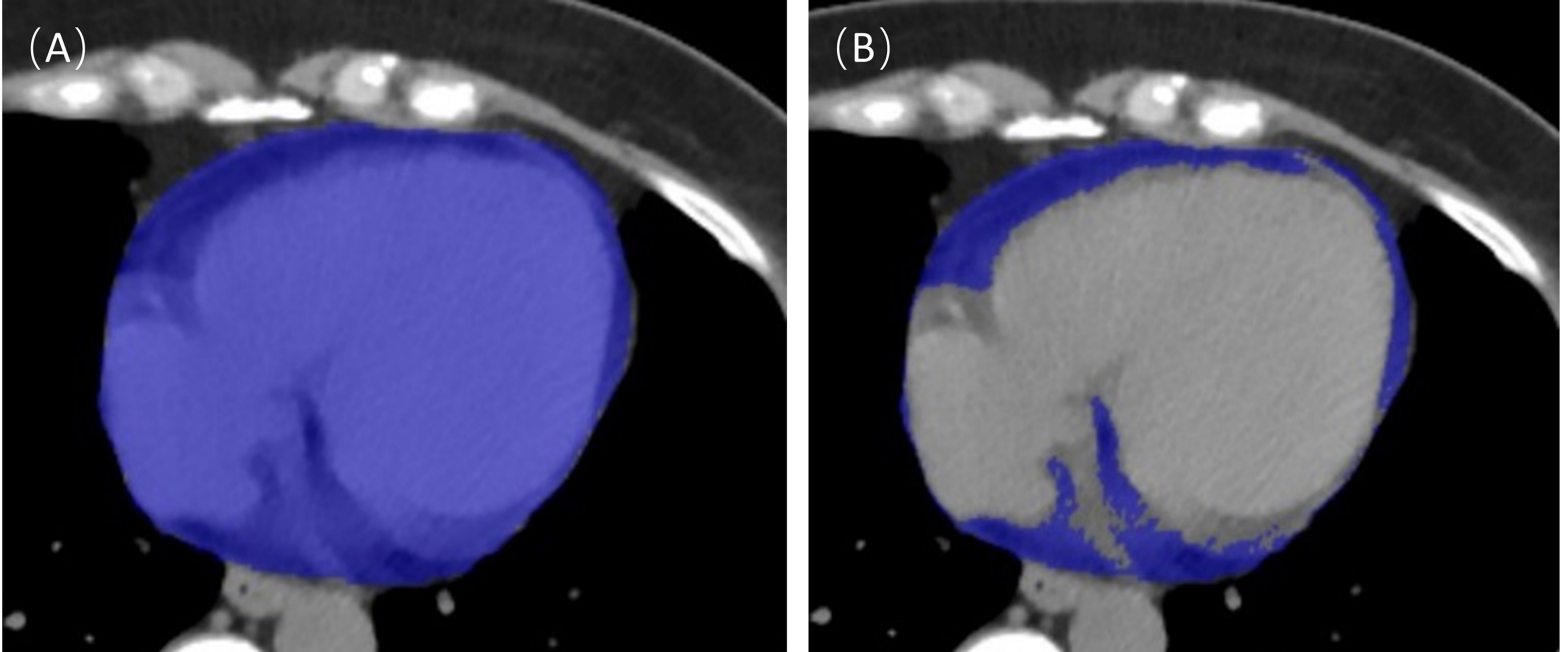
**Epicardial adipose tissue (EAT) segmentation.** The entire cardiac tissue was first automatically segmented as a region of interest (A), and then the tissue within the region of interest with a threshold of –190~–30 Hounsfield units (HU) was extracted and defined as EAT (B).

Quantitative analyses of EAT were conducted using semi-automated software (United Imaging uAI research portal 20230715 system, Shanghai United Imaging Intelligence, Co., Ltd., China). EAT volume (mL) and mean EAT radiodensity (Hounsfield units, HU) were extracted and automatically calculated. Initially, the pericardial region in axial slices from the bifurcation of the lung trunk to the diaphragm was outlined as the region of interest using semi-automatic outlining. Subsequently, thresholds within the region of interest were set between –190 HU and –30 HU. An example of EAT segmentation is shown in Fig. 2. These studies were performed by two radiologists with 3 and 5 years of experience in cardiac CT, respectively, and agreement was reached through negotiation in case of disagreement. It has been shown that EAT volume is positively correlated with body mass index (BMI) [15] and that BMI changes with the progression of chemotherapy. Therefore, we recorded BMI before and after chemotherapy separately and used the EAT volume index (volume index is defined as the volume (mL) divided by the BMI (mL/kg/$m^{2}$) [16]) as the study variable.

### 2.5 Statistical Analysis

The study data was statistically analyzed using SPSS Statistic 26.0 (IBM-SPSS Statistics, Chicago, IL, USA). Normality was tested for all continuous variables using the Shapiro-Wilks method. Continuous variables are expressed as mean ± standard deviation (normally distributed variables) or median and interquartile range (non-normally distributed variables) and were compared using the student’s *t*-test or Mann-Whitney U test. Categorical variables are expressed as percentages and compared using $\chi^{2}$ or Fisher’s exact test. A comparison of EAT volume index and radiodensity between T0 and T1 was performed using paired samples *t*-test for continuous variables between the two groups. The changes in EAT volume indices and radiodensity between the AC-T group and the AC-TH group were tested using student’s *t*-test or the Wilcoxon test, *p* < 0.05 was considered statistically significant. To ensure the reproducibility of EAT, we selected all patients before treatment to assess inter-observer agreement (assessed by physicians with 3 and 5 years of experience in observational chest CT, respectively), and an interclass correlation coefficient (ICC) was used to evaluate the inter-observer agreement of EAT volume and radiodensity.

## 3. Results

### 3.1 Study Population

A total of 41 BC patients met the inclusion and exclusion criteria for this study. All patients underwent complete surgical resection of BC before receiving adjuvant chemotherapy, and their clinical and imaging data were complete. All patients were female and the mean age at diagnosis of BC was 52.15 ± 7.97 years. Among these patients, 23 received adjuvant chemotherapy with the AC-T protocol (Three of the HER-2-positive patients voluntarily abandoned trastuzumab treatment because of financial problems) and 18 received adjuvant chemotherapy with the AC-TH protocol. Table 1 presents a comparison of the demographic data and clinical characteristics between the two groups. The general data, clinical characteristics and pathological data of the two groups were well matched. None of the patients in either group had hyperlipidemia, and the type of carcinoma was infiltrating ductal carcinoma. Additionally, the LVEF before and after chemotherapy remained within normal levels in both the AC-T group (T0: 67.78 ± 4.72%, T1: 66.57 ± 4.25%) and the AC-TH group (T0: 68.94 ± 6.60%, T1: 65.56 ± 4.19%), and there were no clinical manifestations of chemotherapy-induced CTX.

**Table 1.** **Main characteristics of the study population**.

		Total (n = 41)	AC-T (n = 23)	AC-TH (n = 18)	*p *value
Age (years)		52.15 ± 7.97	53.22 ± 8.37	50.78 ± 7.44	0.337
Body weight (kg)		60.60 ± 8.16	60.61 ± 7.80	60.58 ± 8.83	0.992
BMI (${\mathrm{kg/m}}^{2}$), baseline		24.27 ± 2.99	23.88 ± 2.32	24.75 ± 3.70	0.390
Body weight after chemotherapy		60.71 ± 8.14	60.70 ± 7.82	60.72 ± 8.75	0.992
BMI after chemotherapy		24.30 ± 2.85	23.92 ± 2.28	24.79 ± 3.45	0.364
Diabetes		4 (9.8%)	3 (13.0%)	1 (5.6%)	0.618
Hypertension		4 (9.8%)	3 (13.0%)	1 (5.6%)	0.618
Tumor site					0.732
	Left	17 (41.5%)	9 (39.1%)	8 (44.4%)	
	Right	24 (58.5%)	14 (60.9%)	10 (55.6%)	
Pathological variable					
	ER-positive	25 (61.0%)	13 (56.5%)	12 (66.7%)	0.509
	PR-positive	19 (46.3%)	10 (43.5%)	9 (50.0)	0.678
	HER-2 positive	21 (51.2%)	3 (13.0%)	18 (100.0%)	<0.001
Histological grade					0.879
	I	1 (2.4%)	1 (4.3%)	0 (0.0%)	
	II	24 (58.5%)	13 (56.5%)	11 (61.1%)	
	III	16 (39.0%)	9 (39.1%)	7 (38.9%)	
Right breast radiotherapy		9 (22.0%)	7 (30.4%)	2 (11.1%)	0.270
Echocardiographic findings					
	Baseline LVEF	68.29 ± 5.58	67.78 ± 4.72	68.94 ± 6.60	0.533
	End-chemotherapy LVEF	66.12 ± 4.20	66.57 ± 4.25	65.56 ± 4.19	0.452

AC-T, anthracyclines adjuvant chemotherapy protocol; AC-TH, anthracyclines combined with trastuzumab adjuvant chemotherapy protocol; BMI, body mass index; ER, estrogen receptor; PR, progesterone receptor; HER-2, human epidermal growth factor receptor 2; LVEF, left ventricular ejection fraction; n, numbers.

Twenty-two healthy women participated in two chest CT examinations, conducted 18 ± 5 months apart, as part of a health examination follow-up. The average age of the normal group (50.18 ± 12.47 years) was similar to the age of the BC patients in the AC-T group (53.22 ± 8.37 years, *p* = 0.341) and the AC-TH group (50.78 ± 7.44 years, *p* = 0.853). The BMI of the normal group (23.30 ± 2.03 ${\mathrm{kg/m}}^{2}$) was comparable to the BMI before and after chemotherapy in the AC-T group (23.88 ± 2.32 ${\mathrm{kg/m}}^{2}$, *p = *0.377; 23.92 ± 2.28 ${\mathrm{kg/m}}^{2}$, *p* = 0.346) and in the AC-TH group (24.75 ± 3.70 ${\mathrm{kg/m}}^{2}$, *p* = 0.148; 24.79 ± 3.45 ${\mathrm{kg/m}}^{2}$, *p* = 0.119).

### 3.2 Changes in EAT before and after Chemotherapy

**Fig. 3.**
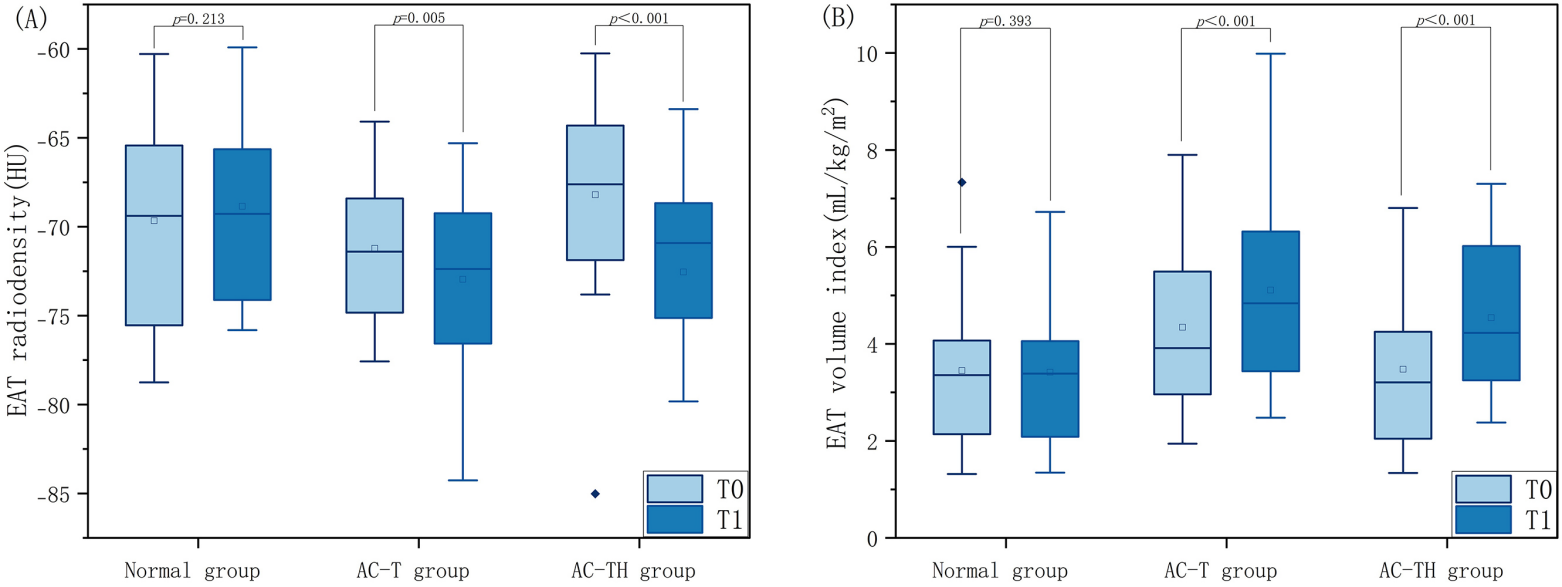
**Comparison of EAT radiodensity and volume index at different timepoints between normal population and patients receiving different chemotherapy protocols. **Distribution of epicardial adipose tissue radiodensity (A) and volume index (B) at baseline (T0) and follow-up (T1) computed tomography examinations in breast cancer patients receiving anthracyclines adjuvant chemotherapy protocol (AC-T) and anthracyclines combined with trastuzumabadjuvant chemotherapy protocol (AC-TH), compared with those of normal healthy women. EAT, epicardial adipose tissue.

In the AC-T group, the EAT volume index significantly increased (5.11 ± 1.85 vs. 4.34 ± 1.55, *p* < 0.001), while the EAT radiodensity was significantly reduced (–72.95 ± 5.01 vs. –71.22 ± 3.91, *p = *0.005) at T1 compared to T0. Similarly, in the AC-TH group, the EAT volume index at T1 significantly increased compared to T0 (4.53 ± 1.61 vs. 3.48 ± 1.62, *p* < 0.001), and the EAT radiodensity at T1 significantly decreased compared to T0 (–72.55 ± 5.27 vs. –68.20 ± 5.98, *p* < 0.001). However, in the normal group, there was no statistically significant difference in EAT volume index and radiodensity between T1 and T0. Table 2 and Fig. 3 summarize the EAT volume index and radiodensity for T0 and T1 and the variations between them.

**Table 2.** **Epicardial adipose tissue volume and radiodensity analysis at baseline and follow-up**.

	T0	T1	*p* value
AC-T (n = 23)			
EAT volume index (mL/kg/$m^{2}$)	4.34 ± 1.55	5.11 ± 1.85	<0.001
EAT radiodensity (HU)	–71.22 ± 3.91	–72.95 ± 5.01	0.005
AC-TH (n = 18)			
EAT volume index (mL/kg/$m^{2}$)	3.48 ± 1.62	4.53 ± 1.61	<0.001
EAT radiodensity (HU)	–68.20 ± 5.98	–72.55 ± 5.27	<0.001
Normal (n = 22)			
EAT volume index (mL/kg/$m^{2}$)	3.45 ± 1.65	3.41 ± 1.59	0.393
EAT radiodensity (HU)	–69.66 ± 5.44	–68.86 ± 5.11	0.213

AC-T, anthracyclines adjuvant chemotherapy protocol; AC-TH, anthracyclines combined with trastuzumab adjuvant chemotherapy protocol; T0, baseline computed tomography; T1, follow-up computed tomography; EAT, epicardial adipose tissue; HU, Hounsfield units; n, numbers.

At T0, the normal group showed similar EAT volume index and radiodensity to the AC-T group (EAT volume index: 3.45 ± 1.65 vs. 4.34 ± 1.55, *p = *0.068; EAT radiodensity: –69.66 ± 5.44 vs. –71.22 ± 3.91, *p* = 0.275) and the AC-TH group (EAT volume index: 3.45 ± 1.65 vs. 3.47 ± 1.62, *p* = 0.957; EAT radiodensity: –69.66 ± 5.44 vs. –68.20 ± 5.98, *p* = 0.424), respectively. At T1, both the AC-T group (5.11 ± 1.85 vs. 3.41 ± 1.59, *p* = 0.002) and the AC-TH group (4.53 ± 1.61 vs. 3.41 ± 1.59, *p* = 0.033) had significantly higher EAT volume index than the normal group. EAT radiodensity was significantly lower in the AC-T group (–72.95 ± 5.01 vs. –68.86 ± 5.11, *p* = 0.009) and the AC-TH group (–72.55 ± 5.27 vs. –68.86 ± 5.11, *p* = 0.031) compared to the normal group.

**Fig. 4.**
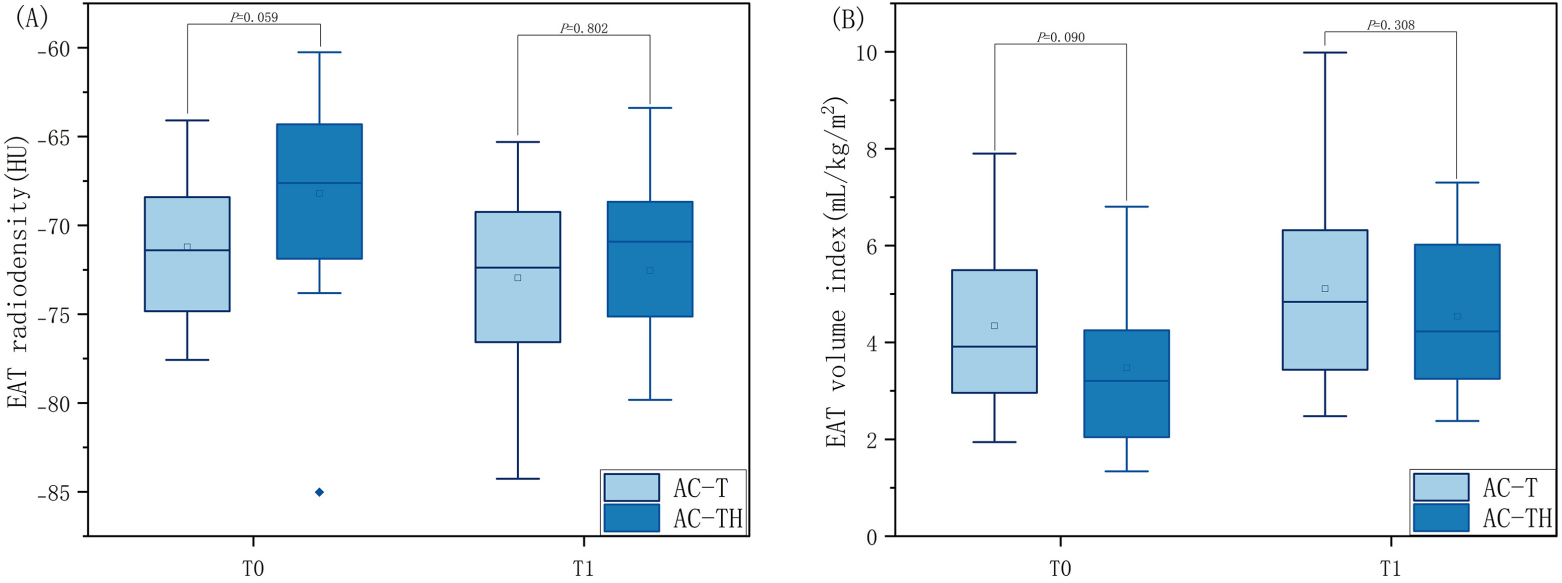
**Comparison of EAT radiodensity and volume index at the same timepoint in patients receiving different chemotherapy protocols. **Distribution of epicardial adipose tissue radiodensity (A) and volume index (B) at baseline (T0) and follow-up (T1) computed tomography examinations in two groups of breast cancer patients receiving adjuvant chemotherapy (i.e., anthracyclines adjuvant chemotherapy protocol (AC-T) or anthracyclines combined with trastuzumabadjuvant chemotherapy protocol (AC-TH)). EAT, epicardial adipose tissue.

To determine whether trastuzumab exacerbates changes in EAT, we also performed further analyses between the AC-T and AC-TH groups. At T0, the difference in EAT volume index (4.34 ± 1.55 vs. 3.48 ± 1.62, *p* = 0.090) and radiodensity (–71.22 ± 3.91 vs. –68.20 ± 5.98, *p* = 0.059) was not statistically significant between patients in the AC-T group and the AC-TH group. Similarly, at T1, there was no significant difference observed in EAT volume index (–5.11 ± 1.85 vs. 4.53 ± 1.61, *p* = 0.308) and radiodensity (–72.95 ± 5.00 vs. –72.54 ± 5.27, *p* = 0.802) between the two groups (Table 3 and Fig. 4). The interobserver agreement for both EAT volume (ICC = 0.896, 95% CI 0.812–0.943) and radiodensity (ICC = 0.856, 95% CI 0.746–0.921) measurements was found to be very good at T0.

**Table 3.** **Comparison of epicardial adipose tissue volume and radiodensity between two different chemotherapy protocols**.

	AC-T (n = 23)	AC-TH (n = 18)	*p *value
EAT volume index (mL/ ${\mathrm{kg/m}}^{2}$)			
T0	4.34 ± 1.55	3.48 ± 1.62	0.090
T1	5.11 ± 1.85	4.53 ± 1.61	0.308
EAT radiodensity (HU)			
T0	–71.22 ± 3.91	–68.20 ± 5.98	0.059
T1	–72.95 ± 5.01	–72.55 ± 5.27	0.802

AC-T, anthracyclines adjuvant chemotherapy protocol; AC-TH, anthracyclines combined with trastuzumab adjuvant chemotherapy protocol; T0, baseline computed tomography; T1, follow-up computed tomography; EAT, epicardial adipose tissue; HU, Hounsfield units; n, numbers.

## 4. Discussion

This study demonstrated that changes in both the volume index and radiodensity of EAT were observed in a group of patients who did not develop CTX, regardless of whether they received AC-T or the AC-TH adjuvant chemotherapy protocol. All patients exhibited an increase in EAT volume index and a decrease in EAT radiodensity. These findings suggest that changes in EAT volume index and density may alert clinicians to focus on BC patients receiving AC-T or AC-TH chemotherapy regimens to prevent the occurrence of CTX.

Under normal conditions, EAT is cardioprotective as a unique fat depot for the heart. It contains more uncoupling protein 1 (UCP-1) and mitochondria, which can produce heat from glucose and free fatty acids. However, in pathological conditions, EAT releases adipokines that are pro-inflammatory and pro-oxidative, which can be detrimental to the heart [8, 17]. It has been well established that estimating EAT using chest CT scans is highly reproducible and shows good agreement with EAT quantified by coronary CT angiography [10, 18]. Therefore, regularly reviewing chest CT images of breast cancer patients allows for detection of EAT and may be of additional prognostic value, especially in patients who have not yet developed CTX.

Trastuzumab and anthracyclines are used as the primary chemotherapeutic agents for BC patients due to their efficacy. However, CTX is a serious complication that can greatly impact the patients’ quality of life and prognosis [2, 4]. Several conventional risk factors, such as advanced age, hypertension, hyperlipidemia, diabetes, and coronary artery disease, may be associated with EAT and may even increase the likelihood of CTX [19, 20]. In this study, the mean age of the patients was 52.15 ± 7.97 years, which falls within the peak age range of 45–54 years for the incidence of BC in Chinese women [11]. There were no significant differences in hypertension and diabetes mellitus between the AC-T and AC-TH groups (*p* = 0.618). Additionally, all patients included in the study were free of hyperlipidemia, and those with a history of coronary artery disease and other cardiac conditions were excluded.

Our study found that the EAT volume index was significantly higher at T1 than at T0 in patients receiving either AC-T or AC-TH adjuvant chemotherapy, which is similar to previous studies by Li *et al*. [3] and Kwon *et al*. [12]. They also found that the EAT volume and the EAT volume index were significantly increased with the use of trastuzumab or anthracyclines. Previous research has shown that increased EAT volume is strongly associated with the progression of coronary artery disease, the occurrence of adverse cardiovascular events, and the prevalence and severity of atrial fibrillation [21, 22]. Moreover, patients with heart failure with preserved ejection fraction (HFpEF) tend to have significantly higher EAT thickness or volume compared to the healthy population. This affects left ventricular function and myocardial substrate utilization, leading to decreased cardiac reserve. In contrast, heart failure with reduced ejection fraction (HFrEF) is often characterized by reduced EAT thickness or volume, which may be related to more severe LV systolic dysfunction, left ventricular remodeling, or lower food intake in critically ill patients [8, 22, 23, 24, 25]. Although they did not experience CTX after chemotherapy, all patients in our study showed a significant increase in EAT volume index. Therefore, we hypothesize that trastuzumab or anthracyclines may increase the EAT volume index as a protective mechanism, resisting early myocardial damage and ventricular remodeling.

In this study, we also observed that the EAT radiodensity at T1 was significantly lower than at T0 in all patients who received adjuvant chemotherapy with AC-T or AC-TH. This finding contradicts the results of two recent studies [3, 12], but aligns with the findings of Monti *et al*. [13]. It is commonly believed that increased EAT radiodensity is associated with higher brown fat content [26] and inflammation and fibrosis in pathological conditions [8, 27]. Conversely, decreased EAT radiodensity is attributed to the conversion of brown fat to white fat, often accompanied by adipocyte hypertrophy and hyperplasia [8, 9, 28]. In this study, several factors may have contributed to the reduced EAT radiodensity in patients receiving adjuvant chemotherapy with AC-T or AC-TH. First, Anthracyclines have the potential to induce oxidative stress by binding to cardiolipin in the inner mitochondrial membrane, thereby increasing the production of reactive oxygen species. Additionally, trastuzumab may exacerbate anthracycline-induced oxidative stress by inhibiting the HER-2 pathway, which would further increase the accumulation of reactive oxygen species, leading to cardiomyocyte death and a progressive increase in myocardial fibrosis [7, 29, 30]. The rise in reactive oxygen species and myocardial fibrosis may be closely associated with the conversion of brown fat to white fat, a phenomenon commonly known as ‘EAT whitening’ [9, 31]. Second, all patients in our study received 5-HT3RA and a single dose of dexamethasone for antiemetic purposes before anthracycline chemotherapy. It has been observed during the COVID-19 pandemic that EAT radiodensity was significantly reduced in COVID-19 patients treated with dexamethasone, possibly due to its anti-inflammatory effect [32]. It has been shown that reduced EAT radiodensity is associated with adverse cardiac metabolic events. Liu *et al*. [33] found that reduced EAT radiodensity was associated with an increased risk of readmission and composite endpoints for heart failure in patients with HFpEF. In addition to this, Franssens *et al*. [34] also found that reduced EAT radiodensity was associated with higher coronary artery calcium scores in men at high risk for cardiovascular disease. Therefore, reduced EAT radiodensity in the present study may be associated with a higher cardiometabolic risk after chemotherapy in BC patients.

Due to the different mechanisms of injury between anthracyclines and trastuzumab leading to CTX, the present study compared patients receiving adjuvant chemotherapy with AC-T and AC-TH to investigate the differences in EAT volume index and EAT radiodensity. At T0, there were no statistically significant differences between the two groups for either EAT volume index (*p = *0.090) or EAT radiodensity (*p* = 0.059). Similarly, at T1, there were still no observed differences in EAT volume index (*p* = 0.308) and EAT radiodensity (*p* = 0.802) between the two treatment groups. Similarly, another recent study also found that no statistical differences were observed in extracellular volume before chemotherapy and at 1 and 5 years after the end of chemotherapy between the two groups of patients who were treated with neoadjuvant chemotherapy using anthracyclines and anthracyclines in combination with trastuzumab [30]. This may be due to the fact that anthracycline-induced type I CTX is often considered irreversible and dose-dependent, while trastuzumab-induced type II CTX is often considered reversible, non-dose-dependent, and has a favorable prognosis [35]. However, it has been found that early detection and treatment can lead to reversible changes in type I CTX [36]. In some cases, patients who developed myocardial injury with trastuzumab did not recover, even after discontinuation of the drug [37], especially when anthracycline drugs were used in combination with trastuzumab, potentially leading to a synergistic effect and minimizing the distinction between the two types of CTX [38]. It is currently believed that the mechanism by which anthracyclines induce the development of CTX include: (1) the interaction of anthracyclines with iron, which leads to iron death, and (2) the dysregulation of the transcriptome mediated by the Topoisomerase IIβ-Dox complex expressed only in cardiomyocytes, which work synergistically to exacerbate oxidative stress [39], and (3) the fact that trastuzumab, which is known for its ability to block HER2 in cardiomyocytes, further exacerbates oxidative stress and apoptosis through blocking the HER2 receptor and further exacerbates oxidative stress and apoptosis [40, 41]. Oxidative stress, on the other hand, has a close relationship with EAT, where increased levels of reactive oxygen species and decreased expression of antioxidant enzymes lead to increased inflammation and promotion of cardiomyocyte fibrosis in EAT, which further leads to changes in EAT volume index and radiodensity [3, 8, 27, 31]. Currently, a >10% reduction in LVEF from baseline with an absolute value of <53% is recognized as a diagnostic criterion for CTX [15]. However, the sensitivity and reproducibility of LVEF are poor. Studies have demonstrated that CTX occurs in 6% to 18% of patients treated with anthracyclines after a median follow-up of 9 years [42]. Therefore, the decrease in LVEF may be a delayed process in disease evolution. When a significant decrease in LVEF occurs, significant cardiac damage may have already happened, often causing patients to miss the optimal treatment window [43]. In this study, all the patients did not meet the diagnostic criteria for CTX, but they did show significant changes in EAT volume index and radiodensity. This establishes a correlation between EAT and subclinical CTX, providing an imaging biomarker for monitoring patients with possible CTX.

## 5. Limitations

There are some limitations in our study. First, it was a retrospective study conducted at a single center with a small sample size due to strict inclusion and exclusion criteria. Second, both chest CT and echocardiography were performed at the same time after the completion of chemotherapy, and none of the patients experienced CTX. Therefore, we could not confirm the relationship between CTX occurrence and specific changes in EAT over time. Prior research demonstrated that myocardial velocities decreased at week 5/6 post-injection of anthracyclines, returning to baseline by week 10, indicating an acute response followed by recovery [44]. Moreover, the delayed LVEF decrease suggests that EAT changes post-chemotherapy might better reflect early CTX changes, but longer follow-up is needed for a comprehensive understanding of EAT evolution in CTX patients. Therefore, we intend to conduct extended follow-up studies to enhance early CTX diagnosis and enable timely risk mitigation by clinicians. Third, the lack of a control group of breast cancer patients not receiving chemotherapy complicates distinguishing disease effects from chemotherapy-induced EAT changes, attributed to limited eligible patients [45]. Note that breast cancer patients without anthracycline and trastuzumab drugs do not show increased heart failure or cardiomyopathy risk [46]. To mitigate confounding factors, we included healthy women as controls. Finally, our study population did not include patients treated with trastuzumab alone. Consequently, we cannot rule out the possibility of a synergistic effect between anthracyclines and trastuzumab on the heart. This makes it difficult to determine whether the changes in EAT caused by type I versus type II CTX differ. The use of semi-automated software improved the reproducibility of EAT measurements, which opens up the possibility of incorporating EAT measurements into routine clinical practice.

## 6. Conclusions

In conclusion, our study found that BC patients undergoing chemotherapy with the AC-T and AC-TH protocols experienced a significant increase in EAT volume index and a significant decrease in EAT radiodensity. Given that breast cancer patients undergo regular follow-up chest CT scans, utilizing these scans to monitor the potential cardiac side effects of anthracyclines and trastuzumab would be an efficient approach without subjecting the patient to additional radiation exposure or financial burden. And the use of semi-automated software improved the reproducibility of EAT measurements, which opens up the possibility of incorporating EAT measurements into routine clinical practice.

## Data Availability

The datasets used and analyzed during the current study are available from corresponding or first author on reasonable request.
